# Biochemical Features and Physiological Roles of hNP22 in the Central Nervous System

**DOI:** 10.3389/fcell.2021.634710

**Published:** 2021-03-04

**Authors:** Ji Wu, Yun-Yi Wang, Xi-Wen Yang, Xiao-Tian Zhang, Jia-Yi Tang

**Affiliations:** ^1^Shanghai Changning Tianshan Traditional Chinese Medicine Hospital, Shanghai, China; ^2^University of Oxford, Oxford, United Kingdom; ^3^Shanghai Literature Institute of Traditional Chinese Medicine, Shanghai, China; ^4^Shuguang Hospital Affiliated to Shanghai University of Traditional Chinese Medicine, Shanghai, China

**Keywords:** hNP22, actin filament, microtubules, neural differentiation, neural plasticity, central nervous system

## Abstract

hNP22, a novel neuron-specific protein that interacts with both actin filaments and microtubules, was found to be highly homologous to the smooth muscle cell cytoskeleton-associated proteins human SM22α and rat acidic calponin. In recent years, functions of hNP22 such as the promotion of neural differentiation and enhancement of neural plasticity, have been described, as well as potential roles of hNP22 in schizophrenia and alcohol-related brain damage (ARBD). Because of the potential roles of hNP22 in neuronal processes and its potential implications in diseases, hNP22 has emerged as a research target. In this paper, we review the gene structure, possible modifications, and functions of the hNP22 protein, as well as its potential clinical significance. Based on its physical structure and previous studies, we speculate that hNP22 has potential biological functions in neurological disorders, such as schizophrenia and ARBD.

## Introduction

In the developing nervous system, the generation of neuronal circuits is involves the differentiation of neuronal subtypes, which is vital for the subsequent formation of synapses. The migration of neuronal precursors stimulates the growth and differentiation of axons and dendrites of spinal neurons, the key parts of this event. In cell migration and neurite extension, signals from the environment control the actin dynamics and motility of cellular processes (Pape et al., 2008). The calponin family of actin-binding proteins functions in direct interactions with the actin and performs regulatory functions through Rho signaling in different cell types (Gimona et al., 2002).

Human neuronal protein 22 (hNP22), a member of the calponin family of proteins is also known as transgelin3 (tgln3). hNP22 was originally identified in a differential display experiment and was differentially expressed in the superior frontal gyrus (SPG) and primary motor cortex (PMC) in the brains of alcoholic patients (Fan et al., 2001). Sequence analysis revealed an ORF containing 597 nucleotides. Comparison between the sequences of hNP22 and rNP25 showed a 96.9% homology in the 199 amino acid overlap. NP22 was also found to be highly homologous to the human protein SM22α and to rat acidic calponin with 67.7 and 44.9% amino acid overlap, respectively (Fan et al., 2001). In smooth muscle, calponin binds to actin, tropomyosin and calmodulin and interacts with brain microtubules in a Ca^2+^-dependent manner through its interaction with tubulin (Fujii and Koizumi, 1999). As a globular protein, SM22α is exclusively expressed in smooth muscle-containing tissues of adult animals (Camoretti-Mercado et al., 1998). SM22α, also termed transgelin, is a transformation and shape change-sensitive actin-gelling protein (Lawson et al., 1997). The sequences of SM22α and calponin were assembled sharing consensus sequences with hNP22. Thus, it is rational to assume that hNP22 has similar biological characteristics to SM22α and calponin. For example, an interaction with actin was confirmed in calponin and SM22α (Takahashi et al., 1986; Fujii et al., 1997; Fu et al., 2000), and was also found in hNP22 (De las Heras et al., 2007).

In recent years, the roles of hNP22 in the promotion of neural differentiation and the enhancement of neural plasticity, as well as the aberrant expression of hNP22 in diseases such as schizophrenia and ARBD have been described (Fan et al., 2001; Ito et al., 2005). Given its important roles in nervous system development and its potential implications in diseases, hNP22 has emerged as a hotspot, and further studies to obtain better knowledge are very important. In this paper, we review the structural functions and clinical implications of the hNP22 protein, as well as the utility and directions for future studies.

## Structure of TAGLN3 Gene

The human NP22 protein is encoded by the TAGLN3 gene located on chromosome 3q13.2, whereas the other two transgelin isoforms, transgelin-1 and transgelin-2, are encoded by the TAGLN1 gene on chromosome 11q23.3 and the TAGLN2 gene on chromosome 11q23.2, respectively (Jo et al., 2018).

**FIGURE 1:**
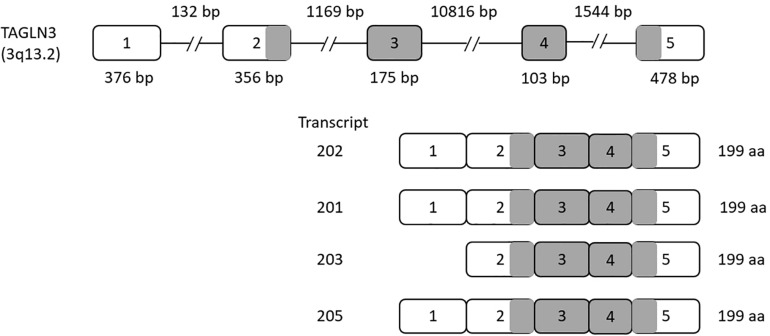
Human Transgelin-3 Gene. Transgelin-3 gene (TAGLN3) structure showing exons (numbered boxes) and introns (line) with their respective sizes. Transcripts 201, 202, 203, and 205 are shown with the gray regions indicating the coding sequence of the transcript.

The TAGLN3 gene contains five exons that give rise to seven different transcripts by alternative splicing. Of these seven transcripts, transcripts 202, 201, 203, and 205 produce a functional protein with 199 amino acids, while transcripts 206, 204, and 207 produce a shorter non-functional protein (as shown in Figure 1).

Orthologs of the TAGLN3 gene have been identified in 258 species, including primates (such as the Bolivian squirrel monkey *Saimiri boliviensis*), rodents (such as the *Chinese hamster Cricetulus griseus*) and fishes (such as the Amazon *Molley Poecilia formosa*). This suggests that the TAGLN3 gene is highly conserved among species. Thus, TAGLN3 might play the same roles in humans as in animal studies.

TAGLN3 was first discovered in the rat brain (Ren et al., 1994). Further functional research in recent years revealed that TAGLN3 could facilitate the neurite outgrowth of chicken dorsal root ganglia (Pape et al., 2008). In this study, TAGLN3 was found to be expressed in the chicken spinal cord, dorsal root, and sympathetic ganglia (Pape et al., 2008). Buchtova et al. (2010) found that TAGLN3 was mainly expressed in the neural crest-derived proximal portion of each cranial ganglion but not in the distal placodal-derived region and the hindbrain. This result revealed that the expression of TAGLN3 might be associated with the onset of neuronal differentiation (Buchtova et al., 2010). In this study, the mediolateral position of TAGLN3-expressing cells in the spinal cord suggests that TAGLN3 is transiently expressed as cells exit the cell cycle. The same roles were also discovered in NP25 in another study (Pape et al., 2008).

## Structure of hNP22 Protein

**FIGURE 2:**
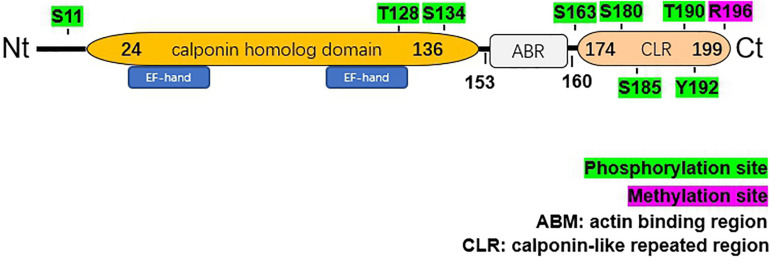
Structural characteristics of human NP22. The hNP22 protein contains an N-terminal calponin-homolog (CH)-domain, an actin binding region (ABR), and a C-terminal calponin-like repeated (CLR)-region. The CH-domain contains two EF-hand motifs. Possible sites for phosphorylation and methylation are labeled in green and pink, respectively.

The 199-amino acid peptide of hNP22 contains an N-terminal calponin homolog (CH)-domain, an actin binding region (ARB) and a C-terminal calponin-like repeat (CLR)-region[citation from ensemble, PROSITE database] (Figure 2).

The C-terminal CLR region is also present in calponin and SM22α. The isolated CLR region failed to associate with F-actin in living cells and did not cosediment with F-actin (Gimona et al., 2002). However, this region might play an important role by manipulating some signaling pathways. Calponin was shown to be involved in the mitogen-activated protein kinase (MAPK) signaling pathway (Menice et al., 1997). In addition, calponin, as a substrate of Rho-kinase, has also been suggested to affect the Rho signaling pathway (Kaneko et al., 2000). Given that hNP22 shares high homology with calponin, especially in the C-terminal CLR region, hNP22 is likely to be involved in these signaling pathways.

Although the C-terminal CLR region identified in the hNP22 gene failed to interact with F-actin, a putative actin binding site (153-R-K-A-Q-Q-N-R-R-160) upstream of the CLR region was identified (Fan et al., 2001). This finding shows the possibility of interactions between hNP22 and actin, which indicates that hNP22 might be a mediator in regulatory signal transduction pathways in neurons.

Two EF-hand Ca^2+^ binding domains (E27-N-K-L-V-D-W-W-I-L-Q-C38 and T107-T-D-I-F-Q-T-V-D-L-W-E118) have been identified within the CH domain, which suggests that the activity of hNP22 might be altered by Ca^2+^ binding. No ATP binding sites or glycosylation sites were identified from the sequence analysis of hNP22 (Fan et al., 2001), indicating that hNP22 is unlikely to be linked to ATP binding or glycosylation. However, a few phosphorylation sites have been identified, suggesting that hNP22 may function through phosphorylation (as shown in Figure 2). PKC phosphorylation sites are also highly conserved in the transgelin/calponin family and it appears that if the serine residue is phosphorylated *in vitro*, actin-binding activity is significantly decreased (Dammeier et al., 2000; Fujii et al., 2000).

## Functions of hNP22

### Interaction Between hNP22 and Actin

The putative actin-binding site of hNP22, suggests that it may regulate the actin cytoskeleton through interaction with actin which might subsequently lead to alterations of various cellular processes, such as the promotion of cell movement and cell differentiation.

Studies on the interactions between hNP22 and actin were carried out in the human neuroblastoma cell line SK-N-SH. Colocalization of rNP25 and F-actin was shown by immunostaining, and a cosedimentation assay further confirmed the association between rNP25 and F-actin. In addition, significant energy transfer between rNP25 and F-actin was measured in the experiment using the fluorescence resonance energy transfer (FRET) technique, indicating close association between NP25 and F-actin (Mori et al., 2004). However, as indirect immunofluorescence was used in the experiment, the result might underestimate the distance between the two molecules. Nevertheless, evidence still favors the interaction between hNP22 and F-actin. The binding of hNP22 and filamentous actin was observed via cosedimentation assays and immunohistology analysis in cultured human neuroblastoma cells (Mori et al., 2004) and Chinese hamster ovary (CHO) cells (De las Heras et al., 2007). Overexpression of NP25 in CHO cells elicited the generation of short cellular processes. Additionally, immunohistochemistry revealed the colocalization of rNP22 and F-actin in neuronal processes in rat brains (Depaz et al., 2005), which further supports the interaction between hNP22 and F-actin.

Immunohistochemistry assays revealed the colocalization of rNP22 and three microtubule markers (tau protein, microtubule-associated protein 2 (MAP2) and alpha-tubulin) in rat brains (Depaz et al., 2005), which shows a possible interaction between rNP22 and microtubules. The immunoprecipitation experiment provides stronger evidence for the binding of hNP22 to tubulin, as they were found to be coprecipitated in both human brain lysate and CHO cells transfected with hNP22 (De las Heras et al., 2007). Indeed, data on the interaction of hNP22 with microtubules are sparse and more studies are needed in the future.

## Clinical Implications of hNP22

As several experiments have shown that hNP22 is a brain-specific cytoskeleton-binding protein, it is assumed that hNP22 might regulate important events in the central nervous system, such as differentiation (especially during process formation and neurite extension) and neural plasticity, via its interaction with actin or its involvement in signal transduction. Dysregulation of hNP22 might be linked to diseases such as ARBD and schizophrenia.

### hNP22 in Neural Differentiation

The initiation of the neural differentiation pathway is regulated by proneural proteins, which accumulate at high levels during early stages of development (Bertrand et al., 2002). However, the expression of proneural genes is downregulated before the cell exits the cell cycle to undergo terminal differentiation. Thus, proneural genes may not initiate neural differentiation directly. Instead, proneural genes might promote neural differentiation by activating some downstream regulatory genes. NeuroM as a downstream gene of proneural genes, is transiently expressed in the spinal cord in cells that have ceased proliferating but have not yet begun to migrate into outer layers. hNP22 and NeuroM showed similar expression patterns, as they were both expressed at early stages during neural development in chick embryos (Pape et al., 2008). Thus, proneural genes function as neural differentiation activators, and hNP22 might act as a downstream effector. These results were consistent with the expression pattern of the TAGLN3 gene.

In a separate study, increased expression of NP25 was discovered both at the mRNA level and at the protein level in rat PC12 cells, a classical model of sympathetic precursor cells, during differentiation induced by nerve growth factor (NGF) (Mori et al., 2004). Increased NP25 expression was also found in mouse neural stem cells during differentiation induced by either 1% fetal bovine serum (FBS) with hepatocyte growth factor (HGF) or 10% FBS. Although the two treatments led to differentiation into different cell types, increased NP25 expression occurred in both cases, which suggests the possible involvement of hNP22 in neural differentiation.

In addition dysregulation of hNP22 expression might be associated with neurodevelopmental diseases, such as schizophrenia. Abormal neuron activation (Carter et al., 1997) and a reduced number of interneurons (Benes et al., 1991) in the anterior cingulate cortex were found to be involved in the pathology of schizophrenia The ratio of hNP22-immunopositive cells/total cells was significantly reduced in layer V and layer VI of the anterior cingulate cortex of the schizophrenic brain compared with age- and sex-matched controls (Ito et al., 2005). A possible interpretation is that the neurological damage observed in schizophrenia might be attributable to the reduced expression of hNP22. However, hNP22 expression in the hippocampus and prefrontal cortex was not significantly different in schizophrenic brains compared with healthy controls (Ito et al., 2005), despite the neuropathological changes observed in the hippocampus and prefrontal cortex in schizophrenic brains (Deicken et al., 2000). Further exploration is needed based on the results from different studies indicating the roles of hNP22 in specific brain regions in schizophrenia.

### hNP22 Promotes Process Formation

hNP22 might be particularly important during process formation. The transfection experiment using CHO cells provides direct evidence. CHO cells transfected with c-myc-hNP22 showed increased hNP22 expression compared with non-transfected cells, and process formation was observed in all transfected cells (De las Heras et al., 2007). The deletion of the putative actin-binding site limited the increase in process formation in transfected CHO cells, which revealed that the binding of actin is involved in the promotion of process formation by hNP22. At the same time, the phosphorylation of hNP22 also alters its ability to promote process formation as the S180A mutation that inhibited the phosphorylation of hNP22 reduced its capacity to enhance process formation and neurite extension (De las Heras et al., 2007).

This reduction in the ability to promote process formation might explain the increased expression of hNP22 found in specific brain regions in ARBD, such as the superior frontal gyrus (SFG) (Kril and Harper, 1989; Fan et al., 2001), prefrontal cortex (PFC) (Depaz et al., 2003) and CA3 and CA4 areas of the hippocampus (Bengochea and Gonzalo, 1990). Long-term ethanol exposure has been shown to cause overactivation of PKC (Stubbs and Slater, 1999), which leads to the phosphorylation of hNP22. And thereby reduces the ability of hNP22 to promote process formation. The upregulation of hNP22 might serve as a compensatory mechanism to compensate for the reduction in the promotion of process formation. However, it is also possible that the increased expression of hNP22 is the cause of the alcohol-induced damage in the SFG and hippocampus. Additional studies could be carried out to examine the link between hNP22 and ARBD.

### hNP22 Regulates Neurite Extension

Neurite extension involves the dynamic reorganization of actin filaments. Neurite elongation and retraction is depend on the balance between the tensile force generated by myosin and actin filaments and the compressive force generated by dynein and microtubules (Ferhat et al., 2001). The neurite length was found to be altered with different levels of NP25 in several cells, which indicated that hNP22 may play a role in the regulation of neurite extension via its interaction with NP25 (Pape et al., 2008). In this study, the overexpression of NP25 led to increased neurite length in PC12 cells and sensory DRG cells (with low endogenous NP25 levels) and to reduced neurite length in chick sympathetic cells (with high endogenous NP25 levels), while the inhibition of NP25 expression by small interfering RNA (siRNA) gave the opposite results. These results show that there exists an optimum NP25 level. Any deviations from the NP25 expression level would inhibit neurite extension. However, the regulation of neurite extension seems to be independent of actin binding as colocalization of NP25 and F-actin was not observed in this experiment. It is possible that hNP22 regulates neurite extension via its interaction with microtubules. On the other hand, the regulation could also occur via the Rho-kinase signaling pathway. Rho GTPases, such as Rnd1 and RhoG, were shown to promote neurite extension (Aoki et al., 2000; Katoh et al., 2000), and they are proposed to work by promoting the hydrolysis of GTP from Rho-associated kinase, thus inhibiting the kinase. Two Rho GTPase-activating proteins (RhoGAPs) that could interact with NP25 have been identified. Thus, it is reasonable to assume that NP25 might activate RhoGAPs and promote neurite extension through the activation of Rho GTPases.

### hNP22 in Neural Plasticity

hNP22 might play a role in synapse rearrangement as an increased level of rNP22 expression was measured in the rat brain by proteomic analysis after a brief period of voluntary exercise, when a high level of synapse rearrangement was expected (Ding et al., 2006). Again, this result provides indirect evidence for the function of hNP22. However, hNP22 does not seem to be involved in the NMDA signaling pathway, which is important in neural plasticity (Gulersen, 2011). Further studies are needed to confirm the possible involvement and understand the underlying mechanism of hNP22 in neural plasticity.

## Conclusion and Prospects

hNP22 is a neural protein that has been shown to interact with both actin filaments and microtubules. It has a CH domain, an actin binding region and a C-terminal CLR region. Two EF-hand Ca^2+^ binding motifs and several phosphorylation sites have been identified on hNP22, suggesting possible regulatory roles of hNP22 via Ca^2+^ binding and phosphorylation. Several pieces of evidence have suggested that hNP22 may play important roles in neural differentiation (especially during process formation and neurite extension) as well as neural plasticity either via its interaction with actin or via its involvement in related signaling pathways. Given its possible physiological roles in the central nervous system, the involvement of hNP22 in neurological disorders, such as schizophrenia and ARBD, has been presumed.

Previous studies have shown increased expression of hNP22 in synaptogenesis, but its functional properties have not been well characterized. In addition, the results of these studies are contradictory. Thus, further studies on the mechanism of hNP22 are required. To gain insight into the biological functions of hNP22, a knockout mouse model would help to provide stronger evidence regarding the functions of hNP22 *in vivo*. Previous studies found the alterations in hNP22 expression in the brain regions where neuropathological changes were observed. Thus, further studies are required to understand whether the alteration of hNP22 expression is a cause or a consequence of neuropathological changes, or both. With a better understanding of the clinical implications of hNP22, it might be used as a new potential therapeutic target for neurological disorders.

In conclusion, several functions and clinical implications of hNP22 have been proposed and studied. As a novel protein, hNP22 was found to be associated with actin, which might play roles in neural differentiation and neural plasticity. Based on all the studies so far, hNP22 has emerged as a protein that shows great potential for exploitation, and further studies to better understand its physiological roles in the central nervous system are required. A greater understanding of the roles and the underlying mechanisms of hNP22 could be beneficial to the development of new treatments for certain neurological disorders.

## Author Contributions

JW and Y-YW conceptualized, wrote, and edited the manuscript. X-TZ collected the literature and prepared the figures. X-TZ, X-WY, and J-YT edited and revised the manuscript. All authors contributed to manuscript revision and read and approved the submitted version.

## Conflict of Interest

The authors declare that the research was conducted in the absence of any commercial or financial relationships that could be construed as a potential conflict of interest.
